# Effect of Chinese herbal medicine injections for treatment of psoriasis vulgaris: a systematic review and meta-analysis

**DOI:** 10.3389/fphar.2023.1148445

**Published:** 2023-07-03

**Authors:** Ziqing Li, Jie Lu, Jiaxin Ou, Jingjie Yu, Chuanjian Lu

**Affiliations:** ^1^ The Second Clinical School of Guangzhou University of Chinese Medicine, Guangzhou, Guangdong, China; ^2^ The Second Affiliated Hospital of Guangzhou University of Chinese Medicine, Guangdong Provincial Hospital of Chinese Medicine and Guangdong Provincial Academy of Chinese Medical Sciences, Guangzhou, Guangdong, China; ^3^ State Key Laboratory of Dampness Syndrome of Chinese Medicine, Guangdong-Hong Kong-Macau Joint Lab on Chinese Medicine and Immune Disease Research and Guangdong Provincial Key Laboratory of Clinical Research on Chinese Medicine Syndrome, Guangzhou, Guangdong, China

**Keywords:** Chinese herbal medicine injections, psoriasis vulgaris, systematic review, meta-analysis, randomized controlled trial

## Abstract

**Background:** Psoriasis vulgaris (PV) is a longstanding, inflammatory, immune-responsive skin condition. Chinese herbal medicine injections (CHMIs) have been utilized for treating PV in Asian countries. This study aims to conduct a thorough systematic review and meta-analysis to comprehensively appraise the efficacy of CHMIs in treating PV.

**Methods:** Seven databases were searched for randomized controlled trials that evaluated the effect of CHMIs in treating PV, ranging from 2004 to June 2022. The meta-analysis was undertaken based on outcome measures, treatment options, and treatment durations using Review Manager 5.4. The primary outcome measure of this study was a 60% or higher reduction in the Psoriasis Area and Severity Index score (PASI 60). A descriptive analysis was performed for the assessment of adverse events.

**Results:** This systematic review incorporated 33 studies, comprising 3,059 participants. The main findings indicated significant differences based on the PASI 60 (RR = 1.30, 95% CI: 1.24 to 1.37, Z = 10.72, *p* < 0.00001), PASI 30 (RR = 1.25, 95% CI: 1.13 to 1.38, Z = 4.48, *p* < 0.00001), and PASI 20 (RR = 1.28, 95% CI: 1.13 to 1.45, Z = 3.82, *p* = 0.0001) outcome measures. Evaluating the treatment options, CHMIs in combination with monotherapies like narrowband ultraviolet B (NB-UVB) and the acitretin capsule (AC) showed a greater reduction in PASI 60 (RR = 1.33, 95% CI: 1.25 to 1.43, Z = 8.32, *p* < 0.00001). In terms of treatment duration, no significant difference was observed when the duration extended beyond 56 days. Furthermore, the results suggested that CHMIs might reduce the incidence of adverse events in the treatment of PV.

**Conclusion:** This systematic review revealed preliminary clinical evidence supporting the use of CHMIs for treating PV, considering outcome measures, treatment options, and treatment durations. However, due to the low methodological quality and limited sample size of the included studies, there is an urgent need for high-quality, multi-center and larger-scale studies of CHMIs for PV to provide robust evidence for their clinical application.

**Systematic Review Registration**: [https://www.crd.york.ac.uk/PROSPERO/display_record.php?RecordID=326531], identifier [CRD42022326531].

## 1 Introduction

Psoriasis vulgaris (PV) is a chronic, inflammatory, immune-driven skin condition characterized by distinct, salmon-pink plaques and silvery scales (Lu et al., 2012; Park et al., 2019; Cave et al., 2020; Griffiths et al., 2021). The global prevalence of PV is between 2.8% and 3.2%, and 0.47% in China, often presenting with numerous comorbidities such as cancer, cardiovascular disease, and mental disorders, which significantly degrade the quality of life of affected individuals (Ding et al., 2012; Rachakonda et al., 2014; Springate et al., 2017). Although the pathogenesis of PV remains incompletely understood, current treatment modalities include topical therapy, phototherapy, and systemic therapy, all of which have shown an increased incidence of adverse events, such as hepatitis and tuberculosis, during long-term use (Armstrong and Read, 2020; Cosio et al., 2021; Doolan et al., 2021; Munera-Campos et al., 2022). Recently, alternative approaches such as Chinese herbal medicine (CHM) have garnered global interest and received mention in the 2021 Joint American Academy of Dermatology–National Psoriasis Foundation guideline due to their prolonged therapeutic effect and lower side effect incidence (Elmets et al., 2021).

Chinese herbal medicine, utilized in clinical practice in Asian countries for centuries, has demonstrated efficacy and safety in alleviating psoriasis symptoms and reducing adverse reactions, as mentioned by numerous studies (Dai et al., 2022; Lu et al., 2022). CHM encompasses various forms, including CHM granules, baths, and injections (CHMIs). Meta-analyses have reported the therapeutic efficacy of CHM granules and baths, thus promoting their international recognition and wider clinical application (Yang et al., 2015; Zhang et al., 2016; Lei et al., 2021). CHMIs, an efficacious form of CHM, includes Buguzhi, Danshen, Huangqi, Xiyanping, and Qingkailing injections, all of which are primarily composed of Chinese herbs. Published studies have demonstrated that CHMIs can mitigate psoriatic symptoms of thick scales, erythema, and pruritus by decreasing the Psoriasis Area and Severity Index (PASI) clinical symptom score (Lu et al., 2011; Ha, 2013; Wang, 2020). However, a comprehensive evaluation of the evidence concerning CHMIs’ effect on PV is currently lacking, hindering the clinical application of CHMI and the diversification of effective PV treatments. Thus, it is imperative to conduct a systematic review and meta-analysis evaluating CHMIs’ efficacy in treating PV, following the standard guidance of the Cochrane Handbook for Systematic Reviews of Interventions and the Preferred Reporting Items for Systematic Review and Meta-Analysis (PRISMA). This will provide clinicians with a new therapeutic option for treating PV and potentially influence future research directions.

## 2 Materials and methods

The protocol for this systematic review and meta-analysis has been registered on the Prospective Register of Systematic Reviews (registration number: CRD42022326531). The study was conducted in accordance with the Cochrane Handbook for Systematic Reviews of Interventions and the Preferred Reporting Items for Systematic Review and Meta-Analysis (PRISMA) guidelines.

### 2.1 Eligibility criteria

All studies assessing the effect of CHMIs on PV were considered for inclusion in this study. Detailed information regarding the inclusion and exclusion criteria is shown in Table 1.

**TABLE 1:** Eligibility criteria for this systematic review.

	Inclusion	Exclusion
Participant	Patients diagnosed with psoriasis vulgaris. No limitations relating to gender, age, race, economic status, or education	Patients without psoriasis vulgaris
Intervention	Patients with psoriasis vulgaris have received CHMIs interventions, such as Buguzhi injection, Danshen injection, Huangqi injection, and Qingkailing injection	Patients with psoriasis vulgaris have not been treated with CHMIs, or the studies’ findings did not wholly reflect the clinical effect of CHMIs
	Patients could be treated with CHMIs alone or combined with other conventional therapies (phototherapy, acitretin capsule, etc.) in the intervention group	
	In addition, the therapy could involve no treatments, placebo, or conventional therapies in the control group	
Outcome	The primary outcome of this study is the Psoriasis Area and Severity Index (PASI) score, and the secondary outcome includes the effective rate and the adverse effects	Studies with incomplete data or data errors
Study design		• Observational studies
		• Reviews
		• Consensus-based studies
	• Randomized controlled trials	• Commentaries
		• Conference abstracts
		• Case reports
		• Animal or cell experiments

### 2.2 Search strategy

**FIGURE 1:**
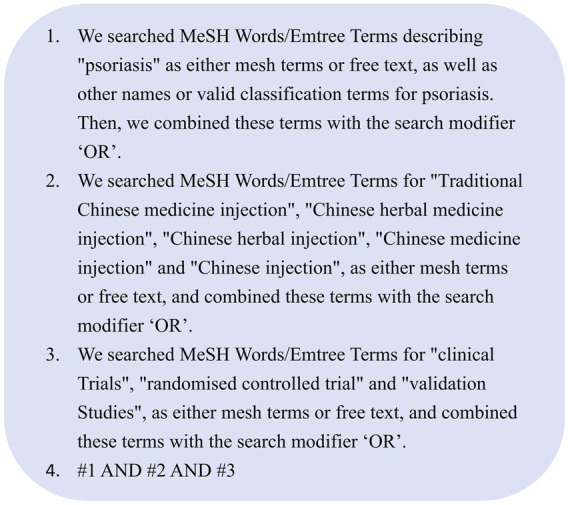
Rearch strategies of systematic review and meta-analysis.

Seven databases were searched from their inception to June 2022, with language restrictions for English and Chinese. These databases include PubMed, Embase, the Cochrane Library, the China National Knowledge Infrastructure (CNKI), the Chinese Biomedical Literature Database (CBM), the Chinese Scientific Journals Database (VIP), and Wanfang Database. The initial search strategy for the databases is shown in Figure 1. In addition to database search, we concurrently explored other resources pertinent to this study, such as reference lists of studies, relevant research reports, and recently registered trials, to ensure comprehensive coverage of relevant data on the effect of CHMIs on PV.

### 2.3 Study selection and data extraction

All retrieved articles from the seven databases were imported into EndNote. Two researchers (ZL and JL) independently screened the search results. Following the removal of duplicates, we obtained the full texts of the remaining studies. Subsequently, all article titles were manually checked to eliminate any overlooked duplicates by EndNote. We then filtered the titles and abstracts that met the eligibility criteria. Full-text articles were then reviewed, and relevant data were extracted using a pre-designed data extraction form. Characteristics of the included studies, such as title, publication year, first author, participants, sample size, age, therapeutic intervention, treatment duration, follow-up duration, and outcome measures, were extracted. Any discrepancies were resolved by a third researcher (CL), and the extracted data were cross-verified by two other researchers (JY and JO).

### 2.4 Risk of bias assessment

Two researchers (ZL and JL) independently assessed the risk of bias following the guidelines of the Cochrane Handbook for Systematic Reviews of Interventions. This included six domains: a) adequate sequence generation, b) allocation concealment, c) blinding, d) handling of incomplete outcome data, e) selective outcome reporting, and f) any other biases. Each domain was categorized as “low risk,” “high risk,” or “unclear risk” in terms of bias risk.

### 2.5 Statistical analysis

Review Manager 5.4 software was utilized to analyze the efficacy of CHMIs for PV. Dichotomous data were expressed using relative risk (RR), while continuous data were represented using mean difference (MD) and the accompanying 95% confidence interval (95% CI) for each effect estimate. The heterogeneity of the included data was evaluated using the chi-squared (X^2^) and the I^2^ tests. Additionally, if substantial heterogeneity was observed, subgroup analysis or publication bias assessment was conducted to explore the potential sources of heterogeneity. Due to the discordance between the frequency count and person–time count when calculating adverse events, a descriptive analysis was preferred for assessing adverse events.

## 3 Results

### 3.1 Study screening results

**FIGURE 2:**
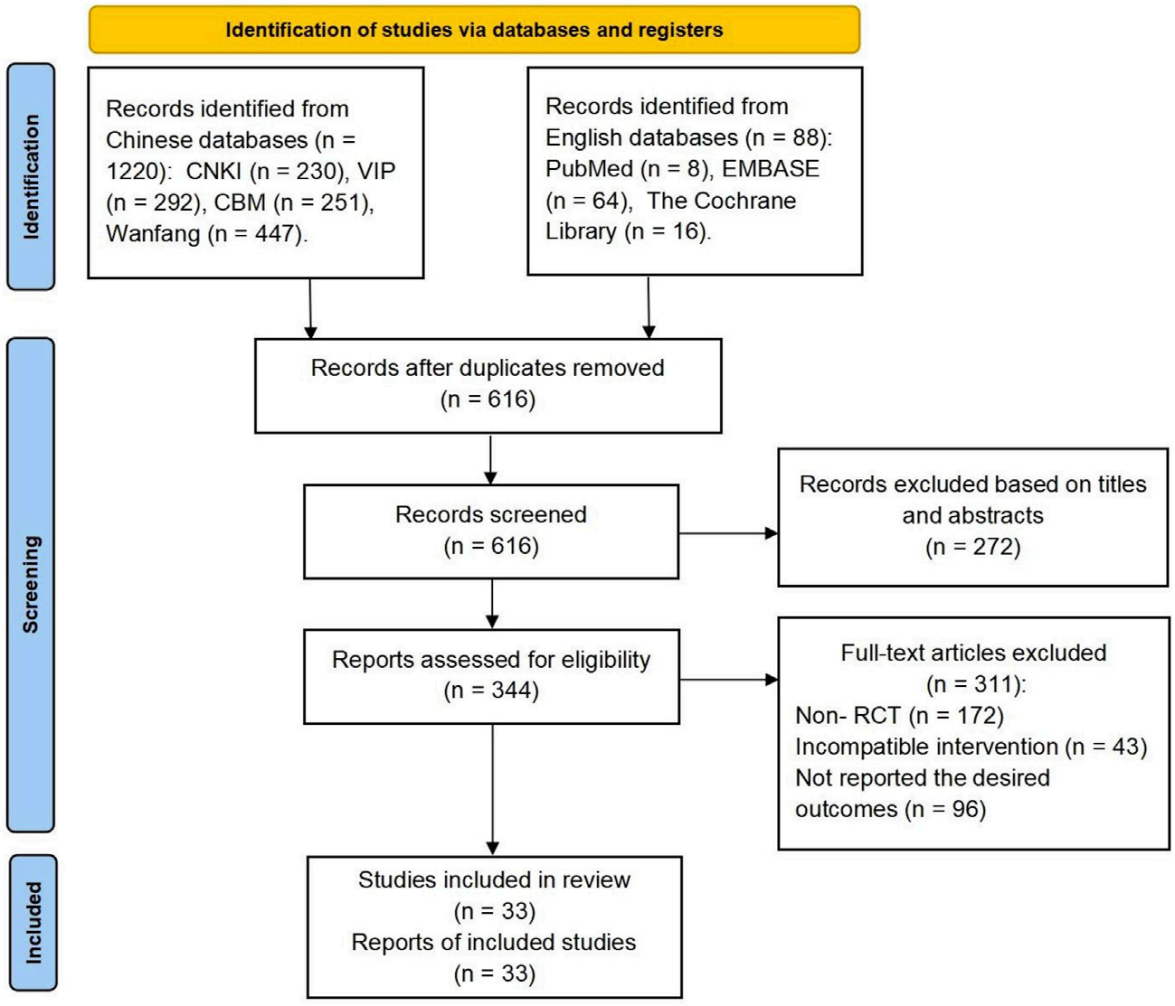
Flowchart for screening eligible studies.

Initially, 1,308 studies were sourced from both English and Chinese databases. Following the removal of duplicates, 616 studies remained and were screened based on their titles and abstracts. Subsequently, 344 eligible studies were meticulously reviewed in their full-text form. Ultimately, 33 pertinent studies were included in this systematic review with their respective references indicated (Pang et al., 2004; Zhong et al., 2004; Li et al., 2006; Hou, 2008; Jiang et al., 2008; Wang, 2008; Zhai et al., 2008; Gu et al., 2009; Li et al., 2009; Liu, 2009; Zhai, 2009; He et al., 2010; Lu et al., 2011; Shen et al., 2011; Tang et al., 2011; Zhao et al., 2011; Ha, 2013; Huo, 2013; Wu et al., 2013; Li et al., 2014; Zhao et al., 2014; Liu et al., 2015; Ma, 2015; Zeng et al., 2015; Bao, 2016; Chen et al., 2016; Guo et al., 2016; Jia et al., 2016; Lu, 2016; Wu, 2016; Lu et al., 2017; Li, 2018; Wang, 2020) (Figure 2).

### 3.2 Characteristics of the included studies

This review incorporated 33 studies involving 3,059 participants, with 1,567 participants in the intervention group and 1,492 in the control group. In these studies, the intervention group received either CHMIs alone or CHMIs combined with conventional therapies, while the control group was treated with either placebo or conventional therapies alone. Among the studies, seven types of CHMIs were used in the intervention group: Buguzhi injection (10 studies) (Li et al., 2006; Zhai et al., 2008; Li et al., 2009; Zhai, 2009; He et al., 2010; Shen et al., 2011; Tang et al., 2011; Ha, 2013; Wu et al., 2013; Ma, 2015), Qingkailing injection (two studies) (Pang et al., 2004; Guo et al., 2016), Danshen injection (six studies) (Zhong et al., 2004; Jiang et al., 2008; Liu, 2009; Huo, 2013; Li et al., 2014; Zhao et al., 2014), Huangqi injection (five studies) (Hou, 2008; Wang, 2008; Gu et al., 2009; Lu et al., 2011; Bao, 2016), Xiyanping injection (seven studies) (Zhao et al., 2011; Zeng et al., 2015; Chen et al., 2016; Jia et al., 2016; Wu, 2016; Li, 2018; Wang, 2020), Reduning injection (two studies) (Lu, 2016; Lu et al., 2017), and Xiangdan injection (one study) (Liu et al., 2015). The characteristics of the included studies are shown in Table 2, and the main ingredients of CHMIs are demonstrated in Table 3.

**TABLE 2:** Characteristics of the included studies.

First author and year	Sample size (T/C)	Intervention		Duration (day)	Outcome index
		T	C		
Ma (2015)	80 (40/40)	Buguzhi injection + NB-UVB	NB-UVB	21	③⑥
Wu et al. (2013)	376 (192/184)	Buguzhi injection + NB-UVB + Keyin pill + coal-tar solution	NB-UVB + Keyin pill + coal-tar solution	30	①
Ha (2013)	86 (43/43)	Buguzhi injection + NB-UVB + hydrocortisone butyrate ointment	NB-UVB + hydrocortisone butyrate ointment	30	①
Shen et al. (2011)	68 (34/34)	Buguzhi injection + NB-UVB	NB-UVB	28	①⑥
Tang et al. (2011)	68 (32/36)	Buguzhi injection + NB-UVB	NB-UVB	42	①
He et al. (2010)	78 (38/40)	Buguzhi injection + AC + Binghuangfule ointment	AC + Binghuangfule ointment	56	①
Li et al. (2009)	60 (30/30)	Buguzhi injection + NB-UVB	NB-UVB	56	①⑥
Zhai (2009)	148 (74/74)	Buguzhi injection + NB-UVB + Vaseline/boric acid ointment	NB-UVB + Vaseline/boric acid ointment	56	①
Zhai et al. (2008)	86 (46/40)	Buguzhi injection + NB-UVB	NB-UVB	28	①⑥
Li et al. (2006)	60 (30/30)	Buguzhi injection + hydrocortisone butyrate ointment + Keyin pill + vitamin E	Hydrocortisone butyrate ointment + Keyin pill + vitamin E	30	①
Guo et al. (2016)	100 (50/50)	Qingkailing injection + Tuhuaiyin + Shidu ointment	Tuhuaiyin + Shidu ointment	28	②
Pang et al. (2004)	67 (32/35)	Qingkailing injection + diammonium glycyrrhizinate injection + glucocorticoid ointment + boric acid ointment + vitamin injection	Diammonium glycyrrhizinate injection + glucocorticoid ointment + boric acid ointment + vitamin injection	28	④
Li et al. (2014)	63 (35/28)	Danshen injection + compound glycyrrhizin injection + AC	Compound glycyrrhizin injection + AC	30	①
Zhao et al. (2014)	62 (32/30)	Danshen injection + compound glycyrrhizin injection	Compound glycyrrhizin injection	15	②
Huo (2013)	68 (40/28)	Danshen injection + blood-letting therapy	Blood-letting therapy	84	①
Liu (2009)	344 (172/172)	Danshen injection + compound *Tripterygium hypoglaucum*	Compound *Tripterygium hypoglaucum*	30	①⑥
Jiang et al. (2008)	58 (32/26)	Danshen injection	Penicillin	56	①
Zhong et al. (2004)	65 (30/35)	Danshen injection + calcipotriol ointment	Calcipotriol ointment	45	②⑥
Bao (2016)	90 (45/45)	Huangqi injection + AC	AC	56	②⑥
Lu et al. (2011)	65 (35/30)	Huangqi injection + NB-UVB	NB-UVB	56	①⑥
Gu et al. (2009)	20 (11/9)	Huangqi injection + AC	AC	NS	①⑥
Wang (2008)	41 (22/19)	Huangqi injection + arotinoid ethylester capsule	Arotinoid ethylester capsule	84–108	①
	66 (37/29)	Huangqi injection + etretin capsule	Etretin capsule		
Hou (2008)	165 (95/70)	Huangqi injection + Liangxue Huoxue formula	Liangxue Huoxue formula	84–108	①
Wang (2020)	78 (39/39)	Xiyanping injection + AC	AC	30	①⑥
Li (2018)	79 (40/39)	Xiyanping injection + AC	AC	14	③
Chen et al. (2016)	66 (33/33)	Xiyanping injection + AC + compound flumetasone ointment	AC + compound flumetasone ointment	30	①
Wu (2016)	78 (36/36)	Xiyanping injection + AC	AC	30	①⑥
Jia et al. (2016)	100 (50/50)	Xiyanping injection + AC	AC	28	③⑥
Zeng et al. (2015)	80 (40/40)	Xiyanping injection + *Tripterygium* glycosides + silicon ointment + triamcinolone and urea cream	*Tripterygium* glycosides + silicon ointment + triamcinolone and urea cream	30	①
Zhao et al. (2011)	48 (26/22)	Xiyanping injection + AC	AC	30	①
Lu et al. (2017)	44 (22/22)	Reduning injection + NB-UVB	NB-UVB	21	⑤
Lu (2016)	60 (30/30)	Reduning injection + Penicillin	Penicillin	15	①
Liu et al. (2015)	48 (24/24)	Xiangdan injection + AC + calcipotriol ointment	AC + calcipotriol ointment	NS	①⑥

T, treatment group; C, control group; ①PASI 60, ②PASI 30, ③PASI 20, ④PASI 70, ⑤PASI 50, and ⑥adverse events; NB-UVB, narrowband ultraviolet B; AC, acitretin capsule; NS, no statement.

**TABLE 3:** Main ingredients of CHMI.

Study	CHMI	Main ingredients
Ma (2015)	Buguzhi injection	Malaytea scurfpea fruit (Buguzhi, *Psoralea corylifolia L*.)
Wu et al. (2013)	Buguzhi injection	Malaytea scurfpea fruit (Buguzhi, *Psoralea corylifolia L*.)
Ha (2013)	Buguzhi injection	Malaytea scurfpea fruit (Buguzhi, *Psoralea corylifolia L*.)
Shen et al. (2011)	Buguzhi injection	Malaytea scurfpea fruit (Buguzhi, *Psoralea corylifolia L.*)
Tang et al. (2011)	Buguzhi injection	Malaytea scurfpea fruit (Buguzhi, *Psoralea corylifolia L*.)
He et al. (2010)	Buguzhi injection	Malaytea scurfpea fruit (Buguzhi, *Psoralea corylifolia L*.)
Li et al. (2009)	Buguzhi injection	Malaytea scurfpea fruit (Buguzhi, *Psoralea corylifolia L*.)
Zhai (2009)	Buguzhi injection	Malaytea scurfpea fruit (Buguzhi, *Psoralea corylifolia L*.)
Zhai et al. (2008)	Buguzhi injection	Malaytea scurfpea fruit (Buguzhi, *Psoralea corylifolia L*.)
Li et al. (2006)	Buguzhi injection	Malaytea scurfpea fruit (Buguzhi, *Psoralea corylifolia L.*)
Guo et al. (2016)	Qingkailing injection	Cape jasmine fruit (Zhizi, *Gardenia jasminoides* Ellis), Isatis root (Banlangen, *Isatis indigotica* Fort.), Nacre (Zhenzhumu, *Hyriopsis cumingii* Lea), Buffalo Horn (Shuiniujiao, *Bubalus bubalis* Linnaeus), Japanese honeysuckle flower (Jinyinhua, *Lonicera japonica* Thunb.), and Baical skullcap root (Huangqin, *Scutellaria baicalensis* Georgi)
Pang et al. (2004)	Qingkailing injection	Cape jasmine fruit (Zhizi, *Gardenia jasminoides* Ellis), Isatis root (Banlangen, *Isatis indigotica* Fort*.*), Nacre (Zhenzhumu, *Hyriopsis cumingii* Lea), Buffalo Horn (Shuiniujiao, *Bubalus bubalis* Linnaeus), Japanese honeysuckle flower (Jinyinhua, *Lonicera japonica* Thunb.), and Baical skullcap root (Huangqin, *Scutellaria baicalensis* Georgi)
Li et al. (2014)	Danshen injection	Danshen root (Danshen, *Salvia miltiorrhiza* Bge*.*)
Zhao et al. (2014)	Compound Danshen injection	Danshen root (Danshen, *Salvia miltiorrhiza* Bge.) and rosewood (Jiangxiang, *Dalbergia odorifera* T. Chen)
Huo (2013)	Danshen injection	Danshen root (Danshen, *Salvia miltiorrhiza* Bge*.*)
Liu (2009)	Danshen injection	Danshen root (Danshen, *Salvia miltiorrhiza* Bge.)
Jiang et al. (2008)	Compound Danshen injection	Danshen root (Danshen, *Salvia miltiorrhiza* Bge.) and rosewood (Jiangxiang, *Dalbergia odorifera* T. Chen)
Zhong et al. (2004)	Danshen injection	Danshen root (Danshen, *Salvia miltiorrhiza* Bge.)
Bao (2016)	Huangqi injection	Milkvetch root (Huangqi, *Astragalus membranaceus* (Fisch.) Bge*. var. mongholicus* (Bge.) Hsiao)
Lu et al. (2011)	Huangqi injection	Milkvetch root (Huangqi, *Astragalus membranaceus* (Fisch.) Bge. *var. mongholicus* (Bge.) Hsiao)
Gu et al. (2009)	Huangqi injection	Milkvetch root (Huangqi, *Astragalus membranaceus* (Fisch.) Bge. *var. mongholicus* (Bge.) Hsiao)
Wang (2008)	Huangqi injection	Milkvetch root (Huangqi, *Astragalus membranaceus* (Fisch.) Bge. *var. mongholicus* (Bge.) Hsiao)
Hou (2008)	Huangqi injection	Milkvetch root (Huangqi, *Astragalus membranaceus* (Fisch.) Bge. *var. mongholicus* (Bge.) Hsiao)
Wang (2020)	Xiyanping injection	Common *Andrographis* herb (Chuanxinlian, *Andrographis paniculata* (Burm. f.) Nees)
Li (2018)	Xiyanping injection	Common *Andrographis* herb (Chuanxinlian, *Andrographis paniculata* (Burm. f.) Nees)
Chen et al. (2016)	Xiyanping injection	Common *Andrographis* herb (Chuanxinlian, *Andrographis paniculata* (Burm. f.) Nees)
Wu (2016)	Xiyanping injection	Common *Andrographis* herb (Chuanxinlian, *Andrographis paniculata* (Burm. f.) Nees)
Jia et al. (2016)	Xiyanping injection	Common *Andrographis* herb (Chuanxinlian, *Andrographis paniculata* (Burm. f.) Nees)
Zeng et al. (2015)	Xiyanping injection	Common *Andrographis* herb (Chuanxinlian, *Andrographis paniculata* (Burm. f.) Nees)
Zhao et al. (2011)	Xiyanping injection	Common *Andrographis* herb (Chuanxinlian, *Andrographis paniculata* (Burm. f.) Nees)
Lu et al. (2017)	Reduning injection	Cape jasmine fruit (Zhizi, *Gardenia jasminoides* Ellis), Japanese honeysuckle flower (Jinyinhua, *Lonicera japonica* Thunb.), and Sweet wormwood herb (Qinghao, *Artemisia annua* L.)
Lu, (2016)	Reduning injection	Cape jasmine fruit (Zhizi, *Gardenia jasminoides* Ellis), Japanese honeysuckle flower (Jinyinhua, *Lonicera japonica* Thunb.), and Sweet wormwood herb (Qinghao, *Artemisia annua* L.)
Liu et al. (2015)	Xiangdan injection	Rosewood (Jiangxiang, *Dalbergia odorifera* T. Chen) and Danshen root (Danshen, *Salvia miltiorrhiza* Bge.)

### 3.3 Risk of bias in the included studies

**FIGURE 3:**
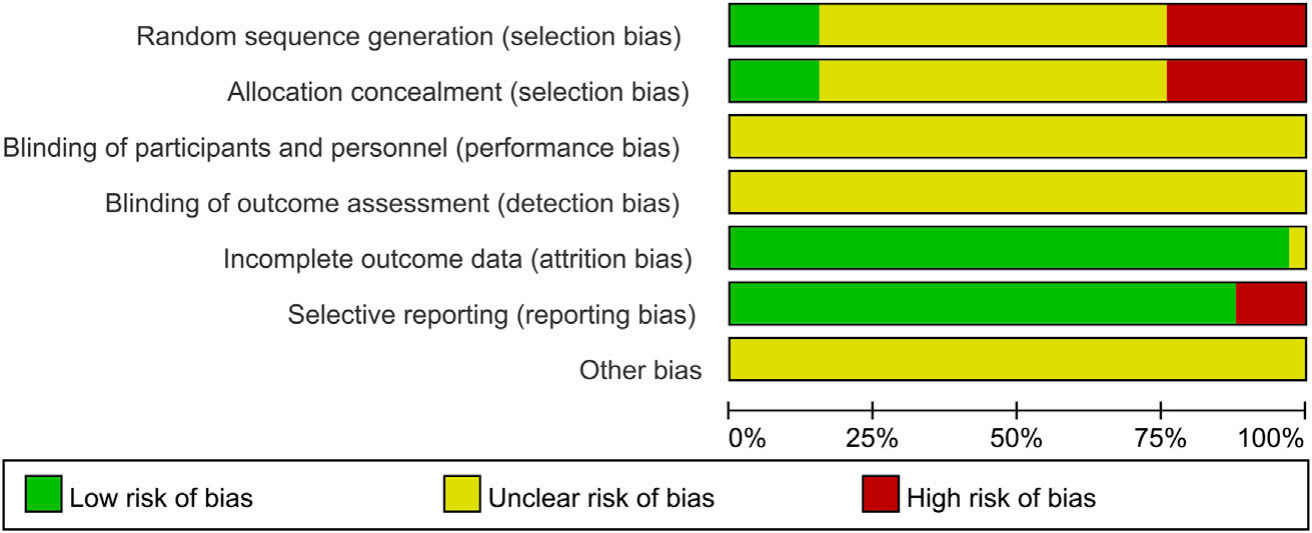
Risk of bias graph for included studies.

**FIGURE 4:**
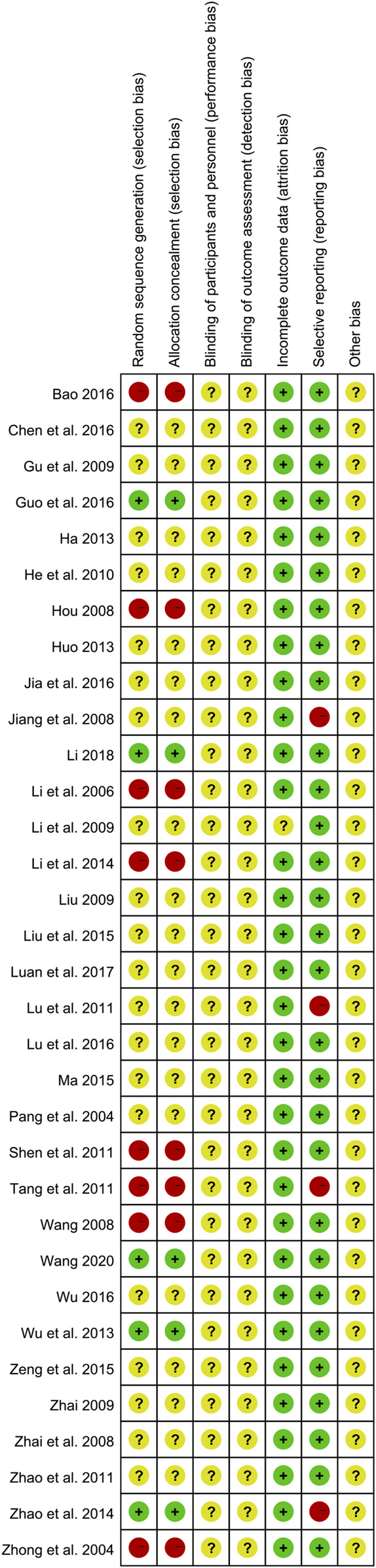
Risk of bias summary for included studies.

The risk of bias assessment results are presented in Figures 3, 4. Among the 33 included studies, only five studies (Wu et al., 2013; Zhao et al., 2014; Guo et al., 2016; Li, 2018; Wang, 2020) utilized random number tables for random sequence generation; hence, their corresponding risk of bias was assessed as low. Eight studies (Zhong et al., 2004; Li et al., 2006; Hou, 2008; Wang, 2008; Shen et al., 2011; Tang et al., 2011; Li et al., 2014; Bao, 2016) were evaluated as having a high risk of bias due to using registration order for randomization. Twenty studies (Pang et al., 2004; Jiang et al., 2008; Zhai et al., 2008; Gu et al., 2009; Li et al., 2009; Liu, 2009; Zhai, 2009; He et al., 2010; Lu et al., 2011; Zhao et al., 2011; Ha, 2013; Huo, 2013; Liu et al., 2015; Ma, 2015; Zeng et al., 2015; Chen et al., 2016; Jia et al., 2016; Lu, 2016; Wu, 2016; Lu et al., 2017) did not specify the method used for randomization, leading to an unclear risk of bias. None of the included studies mentioned blinding of participants and personnel, or blinding of outcome assessors, resulting in an unclear associated risk of bias. One study (Li et al., 2009) reported missing data without providing a detailed reason, leading to an unclear risk of bias. Four studies (Jiang et al., 2008; Lu et al., 2011; Tang et al., 2011; Zhao et al., 2014) failed to fully report the declared variables and were, thus, assessed as having a high risk of bias. As for other biases, none of the studies provided adequate information for risk judgment, resulting in an unclear risk of bias.

### 3.4 Meta-analysis based on outcome measures

The Psoriasis Area and Severity Index (PASI) score is a widely accepted tool to evaluate the severity of PV. This score ranges from 0 to 72, based on factors such as erythema, scaling, and induration (Griffiths et al., 2021). In the included studies, the main outcome measure used to evaluate the effect of CHMIs on PV was PASI 60, defined as a 60% or greater reduction in the PASI score. Other clinical assessment measures included PASI 30 and PASI 20.

#### 3.4.1 Meta-analysis based on PASI 60

**FIGURE 5:**
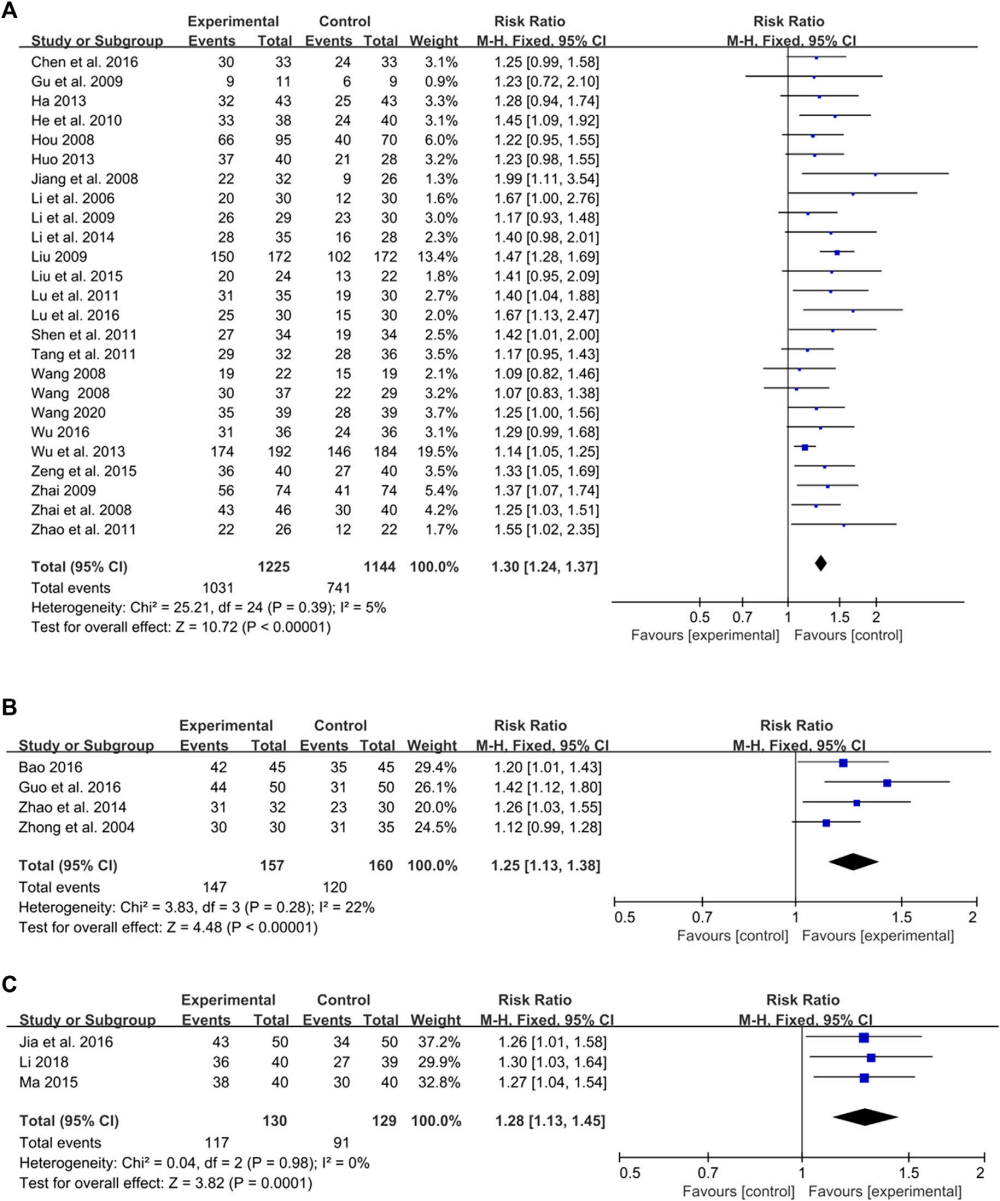
Forest plots based on the PASI score: **(A)** PASI 60; **(B)** PASI 30; **(C)** PASI 20.

Twenty-four studies evaluated the outcome measure of PASI 60, involving 1,225 participants in the intervention group and 1,144 participants in the control group (Li et al., 2006; Hou, 2008; Jiang et al., 2008; Wang, 2008; Zhai et al., 2008; Gu et al., 2009; Li et al., 2009; Liu, 2009; Zhai, 2009; He et al., 2010; Lu et al., 2011; Shen et al., 2011; Tang et al., 2011; Zhao et al., 2011; Ha, 2013; Huo, 2013; Wu et al., 2013; Li et al., 2014; Liu et al., 2015; Zeng et al., 2015; Chen et al., 2016; Lu, 2016; Wu, 2016; Wang, 2020). The heterogeneity test of these studies indicated no significant heterogeneity (*p* = 0.39, I^2^ = 5%), so a fixed-effect model was used for meta-analysis. The result showed a statistically significant difference between the intervention and control groups (RR = 1.30, 95% CI: 1.24 to 1.37, Z = 10.72, *p* < 0.00001), indicating that CHMI was effective in treating PV based on the PASI 60 measure (Figure 5A).

**FIGURE 6:**
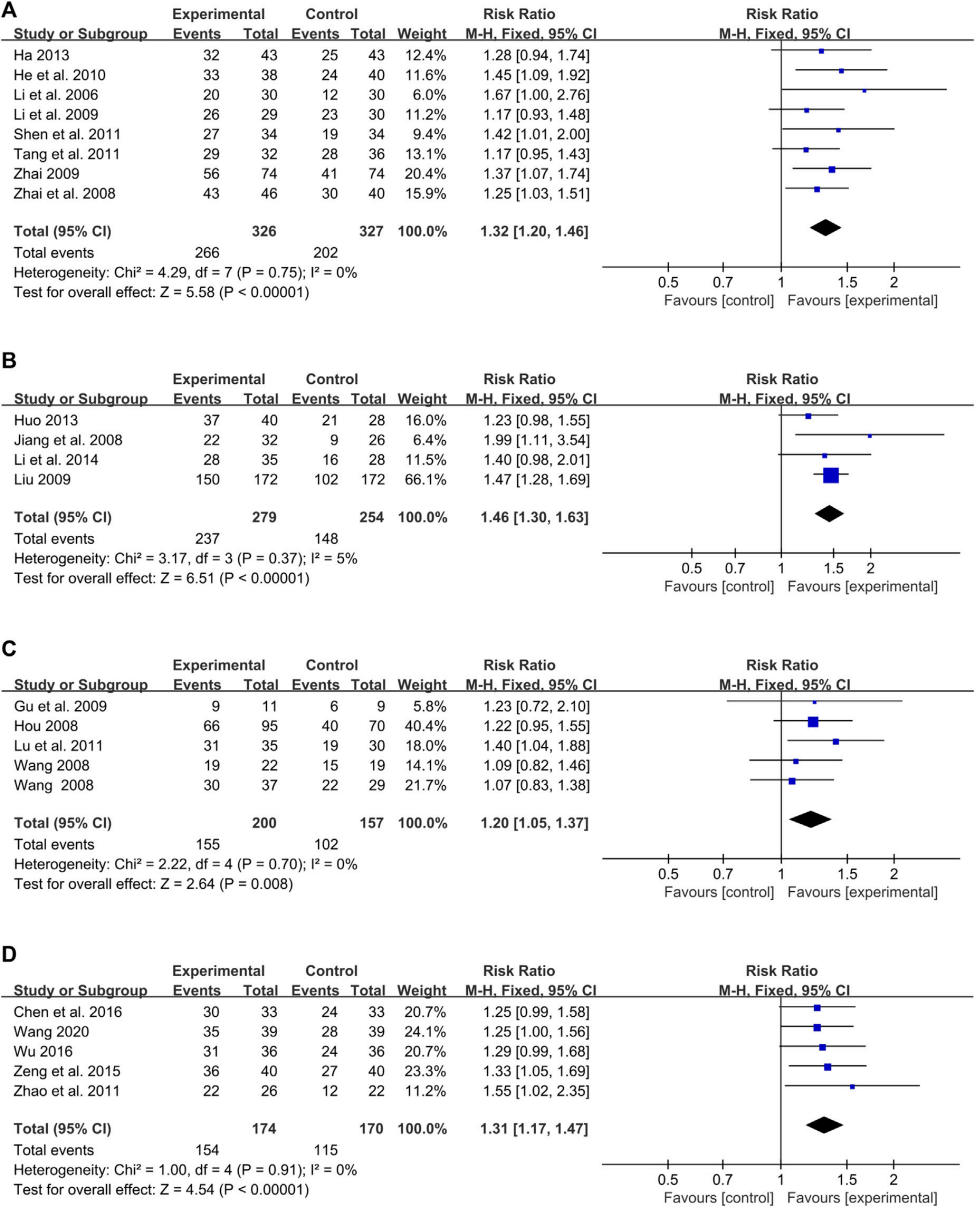
Forest plot of meta-analysis based on PASI 60: **(A)** Buguzhi injection; **(B)** Danshen injection; **(C)** Huangqi injection; **(D)** Xiyanping injection.

Several studies focused on specific types of CHMIs:

• Buguzhi injection: Eight studies mentioned this treatment (Li et al., 2006; Zhai et al., 2008; Li et al., 2009; Zhai, 2009; He et al., 2010; Shen et al., 2011; Tang et al., 2011; Ha, 2013). No significant heterogeneity was found (*P* = 0.75, I^2^ = 0%), and a fixed-effect model was used. The meta-analysis showed a significant difference between the intervention and control groups (RR = 1.32, 95% CI: 1.20 to 1.46, Z = 5.58, *p* < 0.00001) (Figure 6A).
• Danshen injection: Four studies reported on this treatment (Jiang et al., 2008; Liu, 2009; Huo, 2013; Li et al., 2014). No significant heterogeneity was detected (*P* = 0.37, I^2^ = 5%), and a fixed-effect model was used. The result suggested a significant difference between the Danshen injection and control groups (RR = 1.46, 95% CI: 1.30 to 1.63, Z = 6.51, *p* < 0.00001) (Figure 6B).
• Huangqi injection: Four studies evaluated this treatment (Hou, 2008; Wang, 2008; Gu et al., 2009; Lu et al., 2011). A fixed-effect model was selected due to modest heterogeneity (*P* = 0.70, I^2^ = 0%). The result revealed a statistically significant difference between the Huangqi injection and control groups (RR = 1.20, 95% CI: 1.05 to 1.37, Z = 2.64, *p* = 0.008) (Figure 6C).
• Xiyanping injection: Five studies reported on this treatment (Zhao et al., 2011; Zeng et al., 2015; Chen et al., 2016; Wu, 2016; Wang, 2020). No significant heterogeneity was found (*P* = 0.91, I^2^ = 0%), and a fixed-effect model was used. The meta-analysis showed that the Xiyanping injection group had a significant difference compared to the control group (RR = 1.31, 95% CI: 1.17 to 1.47, Z = 4.54, *p* < 0.00001) (Figure 6D).

Overall, these results suggest that different types of CHMIs have a statistically significant impact on the treatment of PV based on the PASI 60 outcome measure.

#### 3.4.2 Meta-analysis based on PASI 30

Four studies, involving 317 participants, reported on the effect of CHMI for PV using PASI 30 as the outcome measure (Zhong et al., 2004; Zhao et al., 2014; Bao, 2016; Guo et al., 2016). With no significant heterogeneity detected (*p* = 0.28, I^2^ = 22%), a fixed-effect model was selected for meta-analysis. The result indicated a significant difference favoring the intervention group (RR = 1.25, 95% CI: 1.13 to 1.38, Z = 4.48, *p* < 0.00001) compared to the control group (Figure 5B).

#### 3.4.3 Meta-analysis based on PASI 20

Three studies, which included 259 participants, used PASI 20 as the outcome measure (Ma, 2015; Jia et al., 2016; Li, 2018). Given the lack of significant heterogeneity (*p* = 0.98, I^2^ = 0%), a fixed-effect model was used for meta-analysis. The findings showed a significant difference between the intervention and control groups (RR = 1.28, 95% CI: 1.13 to 1.45, Z = 3.82, *p* = 0.0001) (Figure 5C).

### 3.5 Meta-analysis based on treatment options

The effect of CHMIs was also evaluated based on different treatment options, with all meta-analyses being conducted using PASI 60 as the outcome measure. Monotherapy was defined as the use of only one kind of therapy when treating PV, such as NB-UVB and AC. Multiple therapies were considered when two or more kinds of therapies were used for PV.

#### 3.5.1 CHMIs plus monotherapy vs. monotherapy

**FIGURE 7:**
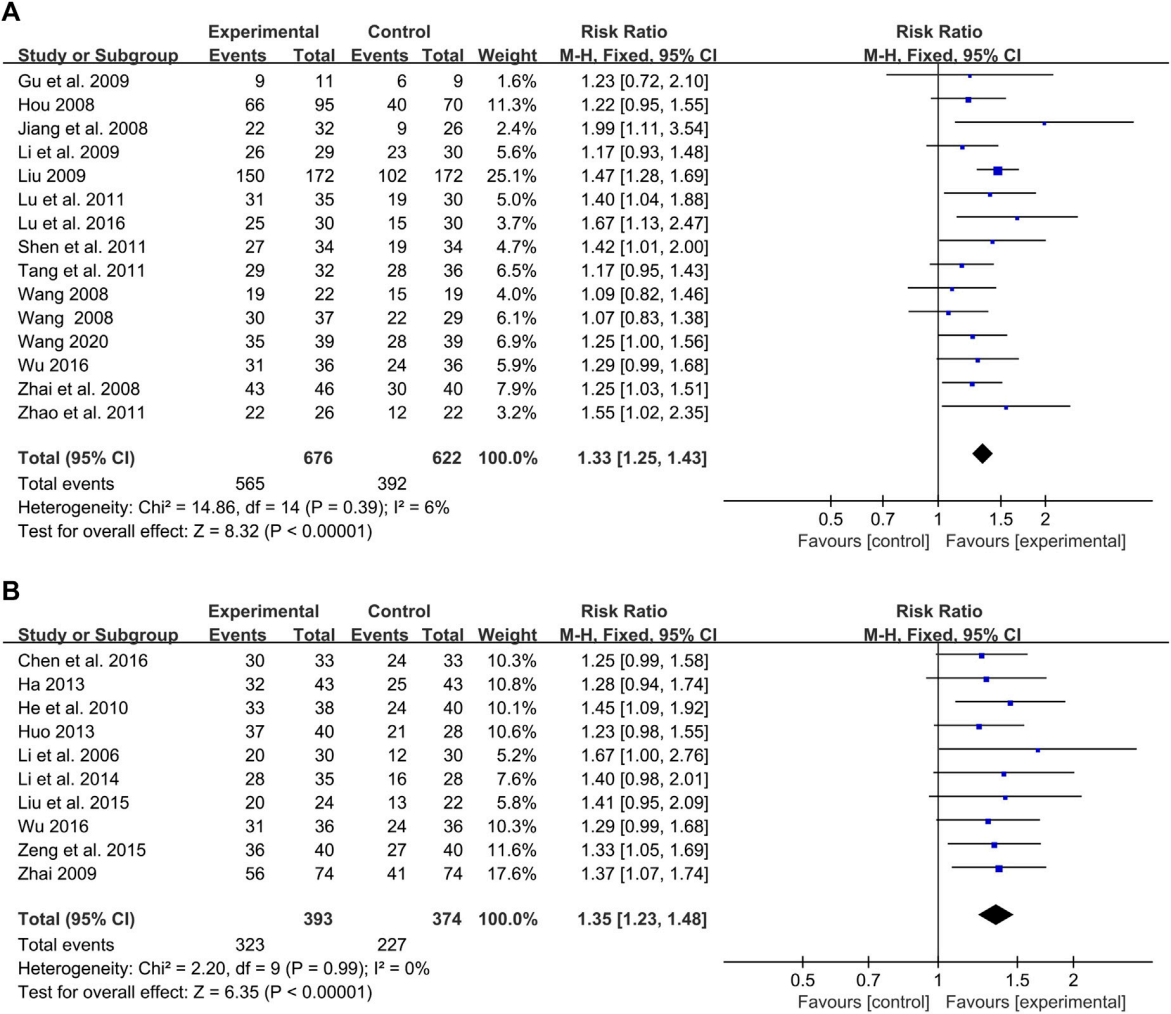
Forest plot of meta-analysis based on treatment options: **(A)** CHMIs + monotherapy vs. monotherapy; **(B)** CHMIs + multiple therapies vs. multiple therapies.

Fourteen studies, involving 1,298 participants, reported on the effect of CHMIs plus monotherapy for PV based on PASI 60 compared to monotherapy used alone (Hou, 2008; Jiang et al., 2008; Wang, 2008; Zhai et al., 2008; Gu et al., 2009; Li et al., 2009; Liu, 2009; Lu et al., 2011; Shen et al., 2011; Tang et al., 2011; Zhao et al., 2011; Lu, 2016; Wu, 2016; Wang, 2020). Given the low heterogeneity (*p* = 0.39, I^2^ = 6%), a fixed-effect model was used for meta-analysis. The result indicated that the combination of CHMI and monotherapy was more effective in treating PV (RR = 1.33, 95% CI: 1.25 to 1.43, Z = 8.32, *p* < 0.00001) compared to monotherapy alone (Figure 7A).

##### 3.5.1.1 CHMIs plus NB-UVB vs. NB-UVB

**FIGURE 8:**
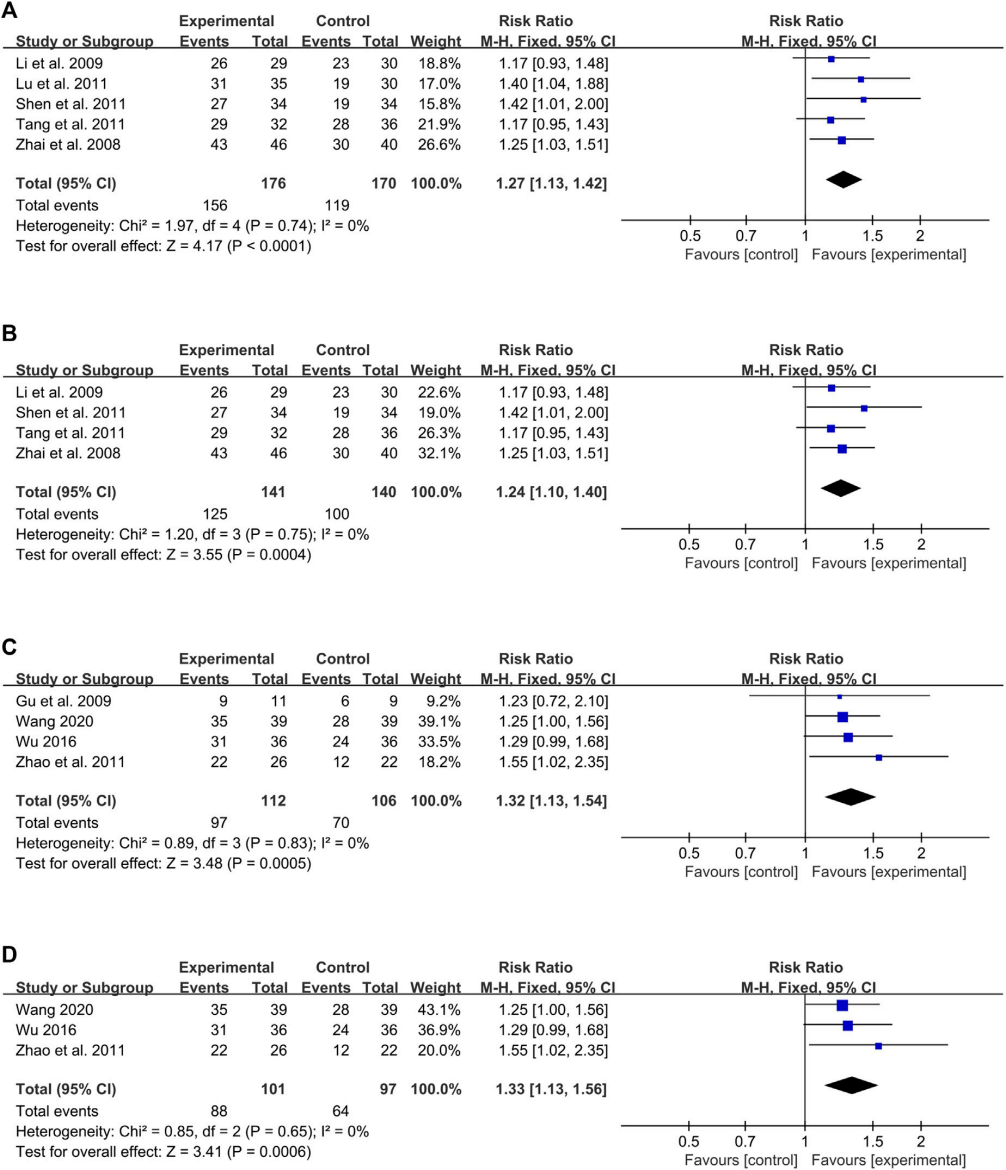
Forest plot of CHMIs + monotherapy vs. monotherapy: **(A)** CHMIs + NB-UVB vs. NB-UVB; **(B)** Buguzhi injection + NB-UVB vs. NB-UVB; **(C)** CHMIs + AC vs. AC; **(D)** Xiyanping injection + AC vs. AC.

Five studies with 346 participants compared the effects of CHMIs plus narrowband ultraviolet B radiation (NB-UVB) to that of NB-UVB alone (Zhai et al., 2008; Li et al., 2009; Lu et al., 2011; Shen et al., 2011; Tang et al., 2011). A fixed-effect model was used for meta-analysis due to no detected heterogeneity (*p* = 0.74, I^2^ = 0%). The finding showed CHMIs plus NB-UVB was more effective than NB-UVB alone in treating PV (RR = 1.27, 95% CI: 1.13 to 1.42, Z = 4.17, *p* < 0.0001) (Figure 8A). Similarly, Buguzhi injection plus NB-UVB was found to be superior to NB-UVB alone (Zhai et al., 2008; Li et al., 2009; Shen et al., 2011; Tang et al., 2011) (RR = 1.24, 95% CI: 1.10 to 1.40, Z = 3.55, *p* = 0.0004) (Figure 8B).

##### 3.5.1.2 CHMIs plus AC vs. AC

Four studies with 218 participants compared CHMIs plus acitretin (AC) to AC alone (Gu et al., 2009; Zhao et al., 2011; Wu, 2016; Wang, 2020), revealing a superior effect of the combination treatment (RR = 1.32, 95% CI: 1.13 to 1.54, Z = 3.48, *p* = 0.0005) (Figure 8C). The combination of Xiyanping injection plus AC was also found to be more effective than AC alone (Zhao et al., 2011; Wu, 2016; Wang, 2020) (RR = 1.33, 95% CI: 1.13 to 1.56, Z = 3.41, *p* = 0.0006) (Figure 8D).

#### 3.5.2 CHMIs plus multiple therapies vs. multiple therapies

Ten studies with 767 participants compared the effects of CHMIs plus multiple therapies to multiple therapies alone (Li et al., 2006; Zhai, 2009; He et al., 2010; Ha, 2013; Huo, 2013; Li et al., 2014; Liu et al., 2015; Zeng et al., 2015; Chen et al., 2016; Wu, 2016). The combination of CHMIs with multiple therapies was more effective (RR = 1.35, 95% CI: 1.23 to 1.48, Z = 6.35, *p* < 0.00001) (Figure 7B).

### 3.6 Meta-analysis based on treatment duration

The meta-analysis was also performed based on the treatment duration of using CHMIs for PV, with the duration of 28, 42, and 56 days.

#### 3.6.1 Duration of 28 days

**FIGURE 9:**
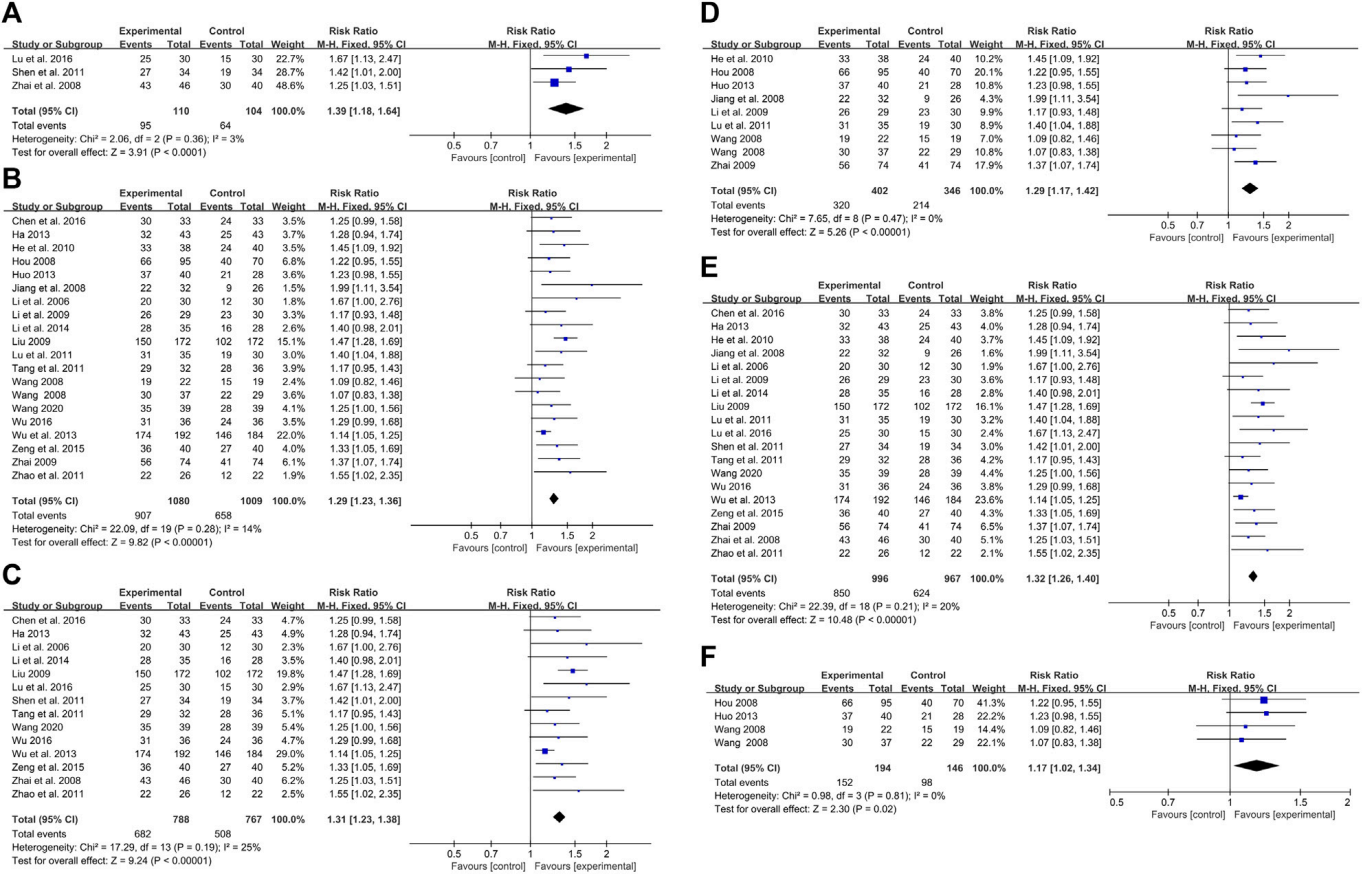
Forest plot based on treatment duration: **(A)** duration 528 days; **(B)** duration>28 days; **(C)** duration 542 days; **(D)** duration>42 days: **(E)** duration 56 days; **(F)** duration >56 days.

Three studies with 214 participants evaluated the effects of CHMIs treatment on PV for up to 28 days (Zhai et al., 2008; Shen et al., 2011; Lu, 2016), showing a significant difference favoring the intervention group (RR = 1.39, 95% CI: 1.18 to 1.64, Z = 3.91, *p* < 0.0001) (Figure 9A). For treatment durations longer than 28 days (Li et al., 2006; Hou, 2008; Jiang et al., 2008; Wang, 2008; Li et al., 2009; Liu, 2009; Zhai, 2009; He et al., 2010; Lu et al., 2011; Tang et al., 2011; Zhao et al., 2011; Ha, 2013; Huo, 2013; Wu et al., 2013; Li et al., 2014; Zeng et al., 2015; Chen et al., 2016; Wu, 2016; Wang, 2020), 19 studies with a total of 2,089 participants were evaluated. The results showed a significant difference favoring the intervention group (RR = 1.29, 95% CI: 1.23 to 1.36, Z = 9.82, *p* < 0.00001) (Figure 9B).

#### 3.6.2 Duration of 42 days

For the duration of 42 days or less, 14 studies with 1,555 participants reported the effect of CHMIs on PV based on PASI 60 (Li et al., 2006; Zhai et al., 2008; Liu, 2009; Shen et al., 2011; Tang et al., 2011; Zhao et al., 2011; Ha, 2013; Wu et al., 2013; Li et al., 2014; Zeng et al., 2015; Chen et al., 2016; Lu, 2016; Wu, 2016; Wang, 2020). A fixed-effect model was used due to detected heterogeneity (*p* = 0.19, I^2^ = 25%). The results showed a significant difference favoring the intervention group (RR = 1.31, 95% CI: 1.23 to 1.38, Z = 9.24, *p* < 0.00001) (Figure 9C). For durations longer than 42 days, eight studies with 748 participants were analyzed (He et al., 2010; Hou, 2008; Huo, 2013; Jiang et al., 2008; Li et al., 2009; Lu et al., 2011; Wang, 2008; Zhai, 2009). The results revealed a significant difference favoring the intervention group (RR = 1.29, 95% CI: 1.17 to 1.42, Z = 5.26, *p* < 0.00001) (Figure 9D).

#### 3.6.3 Duration of 56 days

For the duration of 56 days or less, 19 studies with 1,963 participants were analyzed (Li et al., 2006; Jiang et al., 2008; Zhai et al., 2008; Li et al., 2009; Liu, 2009; Zhai, 2009; He et al., 2010; Lu et al., 2011; Shen et al., 2011; Tang et al., 2011; Zhao et al., 2011; Ha, 2013; Wu et al., 2013; Li et al., 2014; Zeng et al., 2015; Chen et al., 2016; Lu, 2016; Wu, 2016; Wang, 2020). A significant difference was again observed favoring the intervention group (RR = 1.32, 95% CI: 1.26 to 1.40, Z = 10.48, *p* < 0.00001) (Figure 9E). For durations longer than 56 days, three studies with 340 participants were analyzed. Interestingly, the results did not show a significant difference between the CHMIs and control groups (Hou, 2008; Wang, 2008; Huo, 2013) (RR = 1.17, 95% CI: 1.02 to 1.34, Z = 2.30, *p* = 0.02) (Figure 9F).

### 3.7 Descriptive analysis of adverse events

Adverse events associated with CHMIs for PV treatment, including clinical symptomatic and laboratory monitoring adverse events, were described in the included studies. Due to inconsistency in the reporting and frequency of these events, they were not suitable for meta-analysis. Instead, a descriptive analysis was employed (Table 4).

**TABLE 4:** Adverse events of included studies.

Study	Treatment		Sample size	Adverse events											
				Itchi-ness	Dry skin	Dry mouth	Dry eye	Pigmen-tation	Induration at the injection site	Nausea	Loss of appetite	Menstrual disorder	Blood pressure elevation	Transami-nase elevation	Dyslipi-demia
Buguzhi injection															
Ma (2015)	I	Buguzhi injection + NB-UVB	40	—	3	—	—	40	—	—	—	—	—	—	—
	C	NB-UVB	40	—	0	—	—	40	—	—	—	—	—	—	—
Shen et al. (2011)	I	Buguzhi injection + NB-UVB	34	—	—	—	—	34	5	—	—	—	—	—	—
	C	NB-UVB	34	—	—	—	—	34	0	—	—	—	—	—	—
Li et al. (2009)	I	Buguzhi injection + NB-UVB	30	1	—	—	—	1	—	—	—	—	1	—	—
	C	NB-UVB	30	1	—	—	—	0	—	—	—	—	0	—	—
Zhai et al. (2008)	I	Buguzhi injection + NB-UVB	46	3	—	—	—	—	—	—	—	—	—	—	—
	C	NB-UVB	40	2	—	—	—	—	—	—	—	—	—	—	—
Danshen injection															
Liu (2009)	I	Danshen injection + compound *Tripterygium hypoglaucum*	172	—	—	—	—	—	—	1	6	—	—	—	—
	C	Compound *Tripterygium hypoglaucum*	172	—	—	—	—	—	—	0	5	—	—	—	—
Zhong et al. (2004)	I	Danshen injection + calcipotriol ointment	30	2	—	—	—	—	—	—	4	2	—	2	—
	C	Calcipotriol ointment	35	5	—	—	—	—	—	—	4	0	—	1	—
Huangqi injection															
Bao (2016)	I	Huangqi injection + AC	45	—	25	—	28	—	—	—	—	—	—	1	20
	C	AC	45	—	34	—	37	—	—	—	—	—	—	6	32
Lu et al. (2011)	I	Huangqi injection + NB-UVB	35	0	—	—	—	—	—	—	—	—	—	—	—
	C	NB-UVB	30	1	—	—	—	—	—	—	—	—	—	—	—
Gu et al. (2009)	I	Huangqi injection + AC	11	5	—	—	—	—	—	—	—	—	—	—	2
	C	AC	9	7	—	—	—	—	—	—	—	—	—	—	3
Xiyanping injection															
Wang (2020)	I	Xiyanping injection + AC	39	11	7	—	—	—	—	—	—	—	—	3	2
	C	AC	39	13	5	—	—	—	—	—	—	—	—	7	5
Wu (2016)	I	Xiyanping injection + AC	36	9	15	21	—	—	—	—	—	—	—	3	2
	C	AC	36	11	16	23	—	—	—	—	—	—	—	7	8
Jia et al. (2016)	I	Xiyanping injection + AC	50	—	—	12	—	—	—	—	—	—	—	3	3
	C	AC	50	—	—	15	—	—	—	—	—	—	—	9	8
Xiangdan injection															
Liu et al. (2015)	I	Xiangdan injection + AC + calcipotriol ointment	24	—	—	3	—	—	—	—	—	—	—	—	—
	C	AC + Calcipotriol ointment	24	—	—	6	—	—	—	—	—	—	—	—	—

I, intervention group; C, control group; NB-UVB, narrowband ultraviolet B; AC, acitretin capsule; “—”, not reported.

#### 3.7.1 Buguzhi injection

Four studies reported adverse events associated with Buguzhi injection for PV treatment (Zhai et al., 2008; Li et al., 2009; Shen et al., 2011; Ma, 2015). Common clinical symptomatic adverse events included itchiness, skin dryness, and pigmentation. In two studies (Zhai et al., 2008; Li et al., 2009), a total of four cases of itchiness were reported in the Buguzhi injection plus NB-UVB group and three cases in the NB-UVB group. Skin dryness was reported in one study (Ma, 2015), with three cases in the Buguzhi injection plus NB-UVB group. Pigmentation was reported in three studies, with a total of 75 cases in the intervention group and 74 cases in the control group (Li et al., 2009; Shen et al., 2011; Ma, 2015). Induration at the injection site was reported in one study, with five cases (Shen et al., 2011). One study reported a case of elevated blood pressure in the Buguzhi injection plus NB-UVB group (Li et al., 2009).

#### 3.7.2 Danshen injection

Adverse events from Danshen injection for PV treatment were reported in two studies (Zhong et al., 2004; Liu, 2009). Common clinical symptomatic adverse events included itchiness, nausea, loss of appetite, and menstrual disorders. Two cases of itchiness were reported in the intervention group and five cases in the control group in one study (Zhong et al., 2004). A case of nausea was reported in the Danshen injection plus compound *Tripterygium hypoglaucum* group (Liu, 2009). Loss of appetite was reported in two studies with a total of 10 cases in the intervention group and nine cases in the control group (Zhong et al., 2004; Liu, 2009). Menstrual disorders were reported in two cases in the Danshen injection plus calcipotriol ointment group (Zhong et al., 2004). Regarding laboratory monitoring adverse events, elevated transaminase levels were observed in two cases in the intervention group and one case in the control group (Zhong et al., 2004).

#### 3.7.3 Huangqi injection

Three studies reported adverse events associated with Huangqi injection for PV treatment (Gu et al., 2009; Lu et al., 2011; Bao, 2016). Symptomatic adverse events included itchiness, dry skin, and dry eyes. Five cases of itchiness were reported in the intervention group and eight cases in the control group (Gu et al., 2009; Lu et al., 2011). Dry skin was reported in one study, with 25 cases in the intervention group and 34 cases in the control group (Bao, 2016). Dry eyes were reported in one study with 28 cases in the intervention group and 37 cases in the control group (Bao, 2016). As for laboratory monitoring adverse events, elevated transaminase levels were reported in one case in the Huangqi injection plus AC group and six cases in the AC alone group (Bao, 2016). Dyslipidemia was reported in two studies with a total of 22 cases in the intervention group and 35 cases in the control group (Gu et al., 2009; Bao, 2016).

#### 3.7.4 Xiyanping injection

Three studies reported the adverse events related to Xiyanping injection for the treatment of PV (Jia et al., 2016; Wu, 2016; Wang, 2020). In terms of clinical symptomatic adverse events, two studies recorded itchiness in 20 cases in the intervention group and 24 cases in the control group (Wu, 2016; Wang, 2020). Dry skin was reported in 22 cases in the intervention group and 21 cases in the control group (Wu, 2016; Wang, 2020). Two studies also reported dry mouth in 33 cases in the intervention group and 38 cases in the control group (Jia et al., 2016; Wu, 2016). Regarding laboratory monitoring adverse events, transaminase elevation was reported in nine cases in the intervention group and 23 cases in the control group (Jia et al., 2016; Wu, 2016; Wang, 2020). Dyslipidemia was noted in seven cases in the intervention group and 21 cases in the control group (Jia et al., 2016; Wu, 2016; Wang, 2020).

#### 3.7.5 Xiangdan injection

One study reported adverse events associated with Xiangdan injection for PV (Liu et al., 2015). The only clinical symptomatic adverse event reported was dry mouth, which was reported in three cases in the intervention group and six cases in the control group.

## 4 Discussion

### 4.1 Main results of this study

This systematic review aimed to comprehensively and critically evaluate the efficacy of CHMIs in treating PV. A total of 1,308 relevant studies were retrieved, and 33 randomized controlled trials involving 3,059 participants were ultimately included in this review. The findings suggest that CHMIs has significant beneficial effects on PV, as measured by PASI 60, PASI 30, and PASI 20. Regarding treatment options, the meta-analysis revealed that CHMIs in conjunction with monotherapy (UB-UVB and AC) had more PASI 60 responses than monotherapy alone. Specifically, Buguzhi injection plus NB-UVB and Xiyanping injection plus AC demonstrated significantly higher PASI 60 responses than their respective control groups. Additionally, CHMIs combined with multiple therapies showed greater PASI 60 responses than multiple therapies alone.

In terms of treatment duration based on PASI 60, significant differences were detected between the intervention and control groups for durations of 28 and 42 days. However, at the duration of 56 days, a significant difference was only detected for treatment durations of 56 days or less; there was no statistical difference for treatment durations exceeding 56 days.

Regarding adverse events, the review included 13 studies reporting on the adverse reactions of CHMIs for PV treatment. These adverse events fell into two categories: clinical symptomatic (itchiness, dry skin, dry mouth, *etc.*) and laboratory monitoring (transaminase elevation, dyslipidemia, *etc.*) categories. For instance, Buguzhi injection, when used alongside NB-UVB, was associated with pigmentation, possibly due to the reported adverse reactions of NB-UVB in previous studies. Other injections such as Huangqi, Xiyanping, and Xiangdan, when used with AC, frequently resulted in symptomatic adverse events like dry skin, dry eye, and dry mouth, which might be closely related to the application of AC. Interestingly, CHMIs plus AC demonstrated a lower incidence of abnormal laboratory results, such as elevated transaminase and dyslipidemia, compared to AC alone.

Despite these promising results, the mechanisms underlying these effects and adverse events remain unclear and warrant further investigation. Additionally, future research could explore optimal treatment durations and the potential for adverse events related to longer-term use of CHMIs.

### 4.2 Limitations to this study

Despite the rigorous analytical methodology used in this systematic review, several limitations to included studies are worth noting. First, the overall methodological quality of the included studies was low due to incomplete original information, which might lead to an overestimation of the effect of CHMIs for PV treatment. Second, the study primarily used PASI 60 as the main outcome measure, which is not as commonly employed as PASI 50, PASI 75, or PASI 90 in clinical guidelines for PV, potentially impacting the assessment of the included studies. Third, the majority of the included studies were single-center trials with small sample sizes, which might limit the generalizability of the findings. Additionally, there could be a language bias as all studies were sourced from Chinese databases. Finally, the unclear mechanism of CHMIs for PV treatment necessitates further research to better understand the effects and adverse events related to CHMIs.

### 4.3 Implications for clinical practice and future direction

In light of these findings and limitations, several recommendations can be made for future research and clinical practice. First, multi-center studies with larger sample sizes should be conducted to enhance the representativeness and reliability of the results. Second, studies on CHMIs should improve their protocols and emphasize quality control, with a specific focus on the rigorous implementation of randomization, blinding, and allocation concealment. The trials should follow the Consolidated Standards of Reporting Trials (CONSORT) guideline and the latest clinical guidelines for PV.

Finally, experimental research should be carried out to further elucidate the underlying mechanisms of CHMIs for PV. This could increase the evidence base for clinical application and promote the inclusion of CHMIs in international guidelines. The exploration of the optimal dosage, duration of treatment, and the identification of potential adverse events associated with longer-term use of CHMIs could also be potential areas of future research.

## 5 Conclusion

This systematic review has found some promising evidence for the use of CHMIs in the treatment of PV, based on PASI 60, PASI 30, and PASI 20 outcome measures. When compared to monotherapies such as NB-UVB and AC, the combined use of CHMIs with these therapies appears to show an improved therapeutic effect and a more favorable adverse event profile.

However, it is crucial to consider the limitations to the evidence base, notably the low methodological quality and small sample sizes of the included studies. These factors highlight the need for further research into the use of CHMIs for PV. Specifically, we urgently need high-quality, multi-center studies with larger samples to strengthen the evidence supporting the clinical use of CHMIs for PV. This kind of robust research is essential to guide clinical decision-making and to optimize patient care in the future.

## Data Availability

The original contributions presented in the study are included in the article/Supplementary Material; further inquiries can be directed to the corresponding authors.
